# Periprosthetic Joint Infection Caused by *Streptococcus equi* Subspecies *zooepidemicus*: The First Reported Case Confirmed by Synovial Fluid Culture

**DOI:** 10.1155/crdi/3356794

**Published:** 2026-10-28

**Authors:** Marjaana Pitkäpaasi, Goran Varda, Juha O. Grönroos, Elisabet Fellman, Kaisa Huotari, Stergios Lazarinis, Marika Nordberg

**Affiliations:** ^1^ Åland Health Services Mariehamn Åland Islands Finland; ^2^ Infectious Diseases Inflammation Center Helsinki University Hospital and University of Helsinki Helsinki Finland helsinki.fi; ^3^ Department of Clinical Microbiology Turku University Hospital University of Turku Turku Finland utu.fi; ^4^ Veterinary Clinic ElisaVet Jomala Åland Islands Finland; ^5^ Orthopaedics Department of Surgical Sciences Uppsala University Hospital Uppsala University Uppsala Sweden uu.se

**Keywords:** bacteremia, horse exposure, periprosthetic joint infection, *Streptococcus equi* subsp. *zooepidemicus*, zoonosis

## Abstract

**Background:**

*Streptococcus equi* subspecies *zooepidemicus* (SEZ) is a pathogen associated with infections in horses. It can occasionally transmit and cause clinical infections in humans. Even though the bacterium is an opportunistic pathogen, human infections are often severe such as meningitis, septicemia, endocarditis, and septic arthritis.

**Case Report:**

We report a unique case of synovial fluid culture–confirmed periprosthetic joint infection (PJI) caused by SEZ. The patient had undergone a total knee arthroplasty due to osteoarthritis. Five months postoperatively, she was admitted to the hospital with fever and clinical signs of PJI of the knee. The bacterial culture of the synovial fluid and blood cultures revealed SEZ. The 16S rRNA polymerase chain reaction (16S rRNA PCR) and culture of perioperative synovial tissue samples confirmed the same bacterium. Within 48 h of the onset of PJI symptoms, an operation with DAIR (debridement, antibiotics, and implant retention) was performed and followed by 12 weeks of antibiotic therapy. The prosthetic joint was preserved, and the patient had fully recovered with no signs of infection relapse at the 2‐year follow‐up. Two weeks prior to the PJI symptoms, the patient had visited a horse stable, where one of the horses had symptoms of an upper respiratory infection.

**Conclusion:**

We present the first case of synovial fluid culture–confirmed PJI caused by SEZ described in the literature. SEZ can be transmitted from horses to humans and cause serious infections, including PJI. The bacterium should be kept in mind when investigating acute infections, including PJI, in people who encounter horses due to their profession or hobbies.

## 1. Introduction

*Streptococcus equi* subspecies *zooepidemicus* (SEZ) is a Lancefield Group C *β*‐hemolytic streptococcus and belongs to the group of pyogenic streptococci [1, 2]. It is part of the normal bacterial flora of horses and can also cause horse infections, e.g., in the respiratory tract [2]. *Streptococcus equi* subspecies *equi* (SEE) is a host‐restricted streptococcus, found almost exclusively in horses [1], whereas SEZ can cause infections in other animals such as cows, goats, sheep, and pigs [1, 2]. SEZ can also occasionally cause severe infections in humans, such as sepsis, meningitis, endocarditis, septic arthritis, and glomerulonephritis [1, 3–5]. The likely routes of entry of the bacterium into the human body are the respiratory tract, skin, and gastrointestinal tract [6, 7]. Human cases have been associated with exposure to horses or other livestock through direct animal contact during work or leisure activities [1, 3, 5], ingestion of raw meat [2, 8], or contact with horse manure [6]. Consumption of unpasteurized contaminated dairy products can also cause human SEZ infections [4, 9–11].

The aim of this case report is to describe a human periprosthetic joint infection (PJI) caused by SEZ and confirmed by synovial fluid culture, which, to our knowledge, has not been reported previously.

## 2. Case Presentation

A 52‐year‐old healthy female underwent a total knee arthroplasty due to osteoarthritis in the medial compartment of the knee at the surgical department of Åland Health Services in March 2022. The operation was successful, with full recovery and without complications. Postoperative X‐rays showed good alignment of the prosthesis (Figure 1). On the second postoperative day, routine blood tests showed a C‐reactive protein (CRP) level of 92 mg/L (0–12 mg/L), an erythrocyte sedimentation rate of 30 (0–30 mm/h), and a white blood cell count (WBC) of 9.8 × 10^9^/L (3.4–8.2 × 10^9^/L). Four months postoperatively, the patient could return to her usual occupation and regular activities.

**FIGURE 1:**
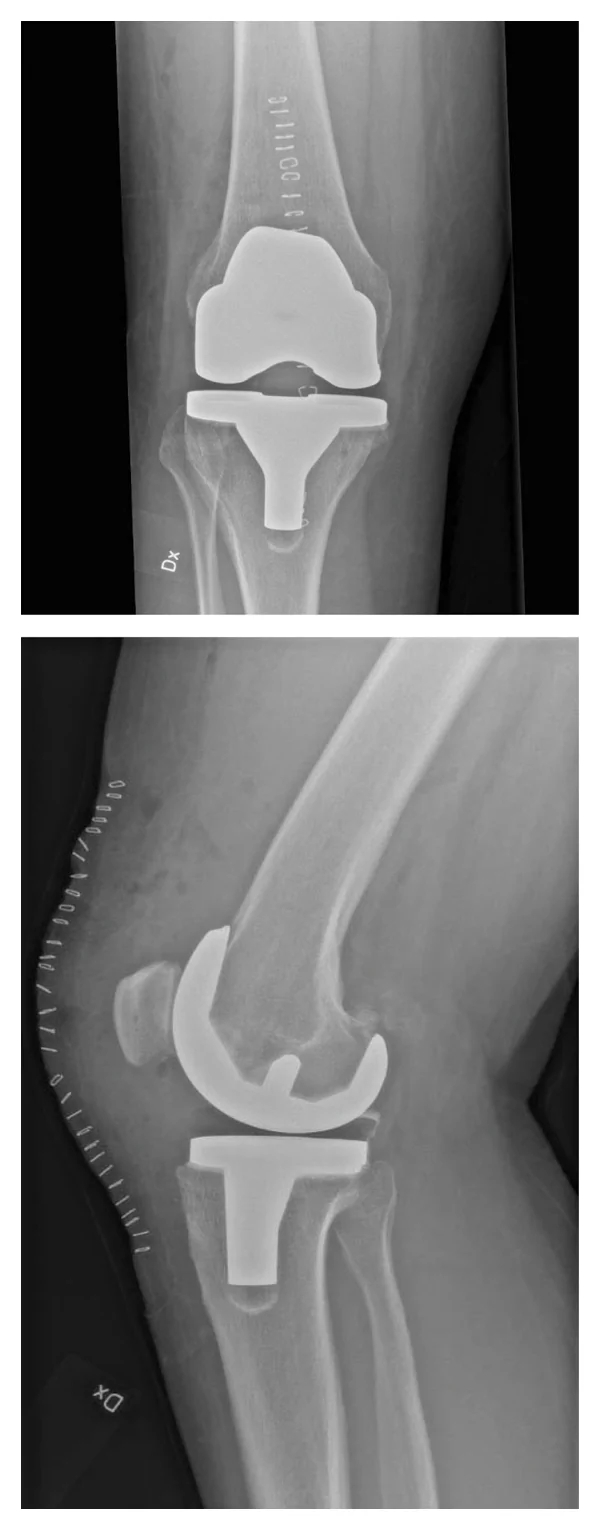
Postoperative knee X‐ray after the total knee arthroplasty showing good alignment of the prosthesis.

In August 2022, the patient was admitted to the Åland Health Services with a 2‐day history of swelling and pain in the operated knee, chills, and nausea. One week prior to the onset of knee symptoms, the patient recalled chills and cervical lymphadenopathy lasting one day.

At the time of admission, all vital signs were stable (blood pressure 127/49 mmHg, heart rate 91 per minute, and respiratory rate 14 per minute), except for a fever up to 38.2°C. Cardiac auscultation revealed no murmurs. The laboratory results showed CRP 296 mg/L, procalcitonin 14.7 *μ*g/L (0–0.5 *μ*g/L), and WBC 22.9 × 10^9^/L. The physical examination of the knee revealed hydrops, local warmth, and pain in palpation and movement but no skin redness.

Knee aspiration was performed, and synovial fluid was sent for cell count, culture, and 16S rRNA PCR analysis. The synovial fluid analysis was consistent with PJI, demonstrating a leukocyte count of 44,587 × 10^6^/L with 81% of polymorphonuclear cells. Lactate was elevated, consistent with a severe inflammatory response (Table 1). The bacterial culture of the synovial fluid and all four blood culture bottles showed Gram‐positive cocci. Both findings were later confirmed to be SEZ by matrix‐associated laser desorption/ionization time‐of‐flight (MALDI‐TOF) mass spectroscopy (Bruker). The 16S rRNA PCR analysis of the synovial fluid also detected the same bacterium. Antimicrobial susceptibility testing was performed with the disk diffusion method according to the European Committee on Antimicrobial Susceptibility Testing (EUCAST) recommendations at the time. The strain was susceptible to benzylpenicillin and therefore also to other relevant beta‐lactams. Resistance was detected to tetracycline and clindamycin. Knee X‐ray showed no signs of implant loosening (Figure 2).

**TABLE 1:** Synovial fluid analysis prior to antimicrobial therapy demonstrating inflammatory response and microbiological confirmation of *Streptococcus equi* subspecies *zooepidemicus*.

WBC, 10^6^/L	44,587
PMN, %	81
Lactate, mmol/L	19.04
Bacterial PCR (16S rRNA)	*Streptococcus equi*
Bacterial culture	*Streptococcus equi* subsp. *zooepidemicus* +++

*Note:* Interpretation according to EBJIS diagnostic criteria for periprosthetic joint infection: synovial fluid WBC > 3000 cells/μL and PMN > 80% are consistent with confirmed periprosthetic joint infection. Elevated lactate is consistent with a severe inflammatory response.

Abbreviations: PCR, polymerase chain reaction; PMN, polymorphonuclear cells; WBC, white blood cell count.

**FIGURE 2:**
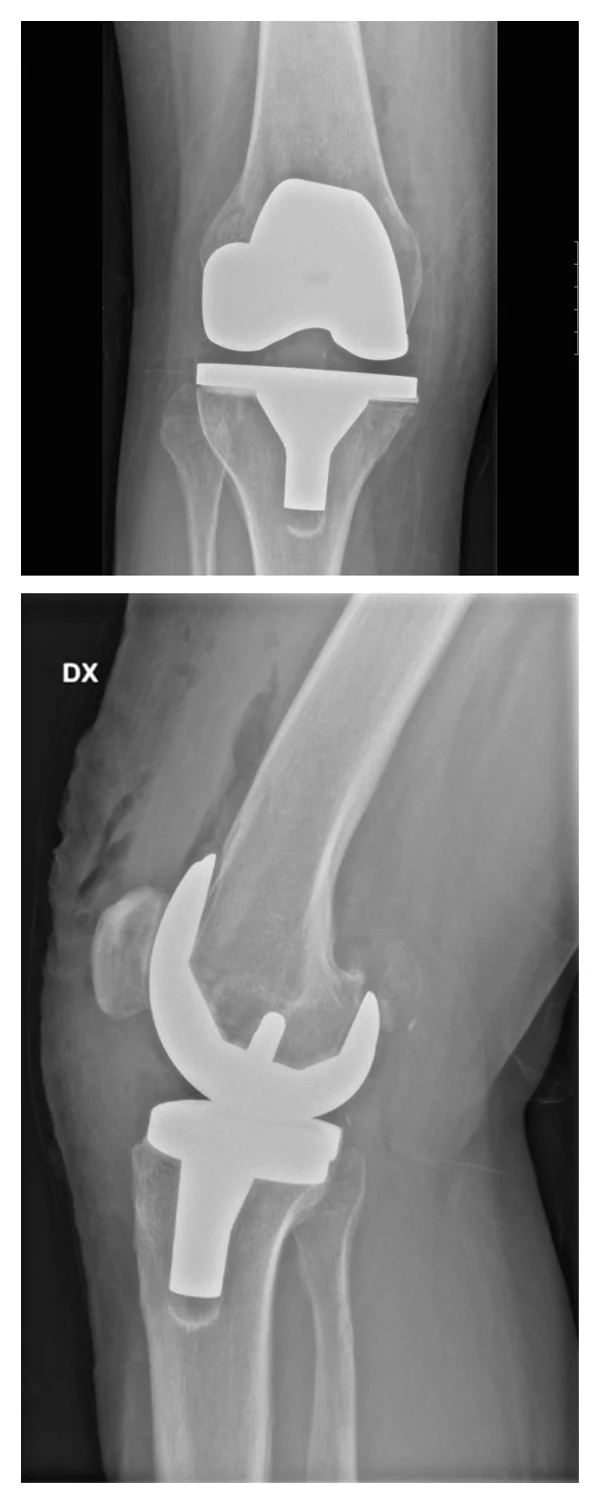
Knee X‐ray following admission for prosthetic joint infection, showing no signs of implant loosening.

The results of the preoperative assessment fulfilled the European Bone and Joint Infection Society (EBJIS) criteria for PJI (Table 1) [12]. The knee implant had no signs of loosening, and a DAIR (debridement, antibiotics, and implant retention) procedure with open debridement and liner exchange was performed the following day. Perioperative tissue and synovial fluid samples were obtained, and SEZ was identified from the samples by culture and 16S rRNA PCR.

After synovial fluid aspiration and blood culture sampling, empirical antibiotic treatment with intravenous cefuroxime was initiated to cover the most common PJI pathogens. The antibiotic treatment was later narrowed to benzylpenicillin at a dose of four million units (MU) six times a day according to the antibiotic susceptibility of the bacterium. The high‐dose regimen was selected because of concomitant bacteremia. Postoperatively, the patient became afebrile and mobilized with full weight bearing on crutches. The local status of the knee improved, and laboratory parameters gradually normalized (Figure 3).

**FIGURE 3:**
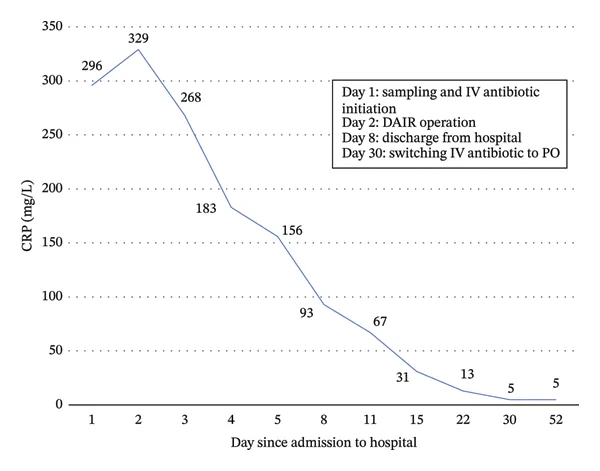
C‐reactive protein (CRP) levels measured during and after hospitalization, demonstrating a rapid decline following initiation of antimicrobial therapy and surgical intervention.

The patient was discharged from the hospital 2 weeks after the DAIR procedure. Benzylpenicillin treatment continued for 2 weeks as a continuous infusion of 20 MU per 24 h using a prefilled infusion pump. After 4 weeks of intravenous antibiotics, oral amoxicillin (1 g, three times daily) was continued for 8 weeks, based on current recommendations for PJI treated with DAIR [13, 14].

After discharge, the patient had regular outpatient follow‐up visits with an orthopedic surgeon and an infectious disease specialist. The symptoms gradually improved, and the patient was pain‐free with slight signs of stiffness with 5 degrees of flexion and extension deficit (−5 to 115°) compared to the range of movement before developing the infection. All laboratory tests were also normal. At the 7‐month follow‐up, the patient had fully recovered and returned to her regular activities.

The patient lives with her family on a horse farm. Other family members usually deal with the daily care of the horses. Two weeks before the onset of the PJI, the patient had exceptionally visited the horse stable and helped with the care of the horses. At the same time, one of the horses had symptoms of an upper respiratory infection. Due to the suspicion of a potentially horse‐related human infection, all the horses on the farm were tested by nasal swabs 3 months after the PJI. The samples were analyzed with 16S rRNA PCR, but all test results were found negative for SEZ.

## 3. Discussion

We report the first case of synovial fluid culture–confirmed PJI caused by SEZ. In our patient, synovial fluid sampling was performed prior to initiation of antimicrobial therapy, allowing microbiological confirmation from both synovial fluid and blood cultures. According to the EBJIS PJI definition, synovial fluid culture obtained prior to antimicrobial therapy remains the gold standard for diagnosing prosthetic joint infections, reliably confirming the presence of the infecting organism within the joint.

In human PJIs, the most frequently isolated organisms are coagulase‐negative staphylococci (30%–43% of cases) and *Staphylococcus aureus* (12%–23%). Mixed microbial flora is found in approximately 10%–11% of cases, while streptococci account for about 10% of PJI pathogens [15]. Human PJIs caused by SEZ are extremely rare, although this organism has been reported to cause severe invasive infections, such as sepsis, meningitis, endocarditis, glomerulonephritis, and septic arthritis [1].

One previous PJI case caused by SEZ has been published [16], in which the diagnosis relied on SEZ growth in blood cultures. Synovial fluid cultures were obtained after surgical washout and remained negative, which does not fulfill the EBJIS definitions of PJI. In that case, only joint washout was performed without adequate surgical debridement, and prolonged antibiotic therapy was used, consisting of intravenous ceftriaxone for 6 weeks followed by oral amoxicillin–clavulanate. Oral therapy was given for 6 months and subsequently extended as suppressive treatment for an additional 12 months.

In addition to the one report of PJI, 13 cases of septic arthritis in native joints have been described across 11 publications [2, 3, 6–8, 10, 11, 17–20]. Among these, three patients had direct horse contact [3, 7, 17]. The most likely routes of infection were respiratory; however, in one case, an unusual route via a chronic plantar wound was suspected [7]. One patient had indirect horse contact via handling horse manure [6]. Six cases were linked to gastrointestinal exposure, including consumption of unpasteurized milk [11], fresh goat cheese [10], raw horse meat [2], or raw pork products [8]. In three cases [18–20], no clear animal or dietary exposure was identified, although one patient had a close relative who owned horses [20]. These reports highlight that SEZ can infect joints via multiple routes. Notably, the previously published PJI case also involved repeated horse contact [16], underscoring the potential zoonotic risk of the organism.

Our patient experienced chills and cervical lymphadenopathy 1 week after the stable visit and 1 week prior to the onset of PJI symptoms, suggesting respiratory transmission and subsequent hematogenous spread, supported by the presence of SEZ in both synovial fluid and blood cultures. The onset of infection coincided with a visit to the stable 2 weeks earlier, during which one of the horses had an upper respiratory infection. The equine samples, which tested negative for SEZ, were collected 3 months after the suspected exposure, which may have reduced the likelihood of detection. The patient reported no other animal contacts or consumption of unpasteurized milk or raw meat. Although SEZ was not detected in the horses at the stable, horse‐associated transmission was suspected.

Delayed PJIs (3–24 months after surgery) are usually caused by less virulent microorganisms, present with subtle signs and symptoms, and are typically acquired during prosthesis implantation [15]. In contrast, our patient’s infection was acquired hematogenously, and the pathogen was both virulent and zoonotic. Due to early DAIR, a shorter course of targeted antibiotic therapy was sufficient to achieve cure and preserve the prosthesis. Our patient received intravenous benzylpenicillin for 4 weeks, followed by oral amoxicillin for 8 weeks, resulting in clinical cure without the need for long‐term suppressive therapy. At the 2‐year follow‐up, the prosthetic joint did not show any signs of loosening, and the patient had completely recovered without signs of infection relapse. This case suggests that early DAIR combined with targeted antimicrobial therapy may be a successful strategy in selected patients with PJI caused by SEZ.

## 4. Conclusion

This case represents the first human PJI caused by SEZ confirmed by synovial fluid culture reported in the literature. Clinicians should be aware that zoonotic pathogens, such as SEZ, can cause various infections, including PJI, in individuals with horse exposure (e.g., veterinarians, riders, and horse owners). Early diagnosis, combined with appropriate surgical and antibiotic management, can result in infection cure and prosthesis preservation.

## Author Contributions

Marjaana Pitkäpaasi, Marika Nordberg, Elisabet Fellman, Stergios Lazarinis, and Goran Varda conceptualized the study. Marjaana Pitkäpaasi drafted the original manuscript. Goran Varda, Marjaana Pitkäpaasi, and Marika Nordberg were involved in clinical management and follow‐up of the patient. Clinical data were collected by Marjaana Pitkäpaasi, Marika Nordberg, Goran Varda, and Juha O. Grönroos. Elisabet Fellman performed the microbiological testing of the horses. Marjaana Pitkäpaasi, Elisabet Fellman, Juha O. Grönroos, Kaisa Huotari, Stergios Lazarinis, Goran Varda, and Marika Nordberg reviewed and edited the manuscript.

## Funding

The authors declare that they have no sources of funding for the preparation of the manuscript.

## Disclosure

All authors read and approved the final manuscript.

## Consent

In accordance with the ethical guidelines for case report publication, written informed consent for publication was obtained from the patient.

## Conflicts of Interest

The authors declare no conflicts of interest.

## Supporting Information

Additional supporting information can be found online in the Supporting Information section.

## Supporting information


**Supporting Information** CARE‐checklist‐SEZ.

## Data Availability

All data generated or analyzed during this study are included in this published article.
